# Cytomegalovirus reactivation during treatment with bispecific antibodies for relapsed/refractory multiple myeloma

**DOI:** 10.1038/s41408-025-01393-8

**Published:** 2025-11-01

**Authors:** Eric Jurgens, Tala Shekarkhand, Colin Rueda, David Nemirovsky, Andriy Derkach, Ross S. Firestone, Kevin Miller, Bruno Almeida Costa, Sridevi Rajeeve, Alexander M. Lesokhin, Neha Korde, Carlyn R. Tan, Hamza Hashmi, Hani Hassoun, Kylee Maclachlan, Urvi A. Shah, Malin Hultcrantz, Issam Hamadeh, Sergio A. Giralt, Gunjan L. Shah, Heather J. Landau, Michael Scordo, Saad Z. Usmani, Sham Mailankody, Zainab Shahid

**Affiliations:** 1 Myeloma Service, Department of Medicine, Memorial Sloan Kettering Cancer Center, New York, NY USA; 2 Department of Epidemiology and Biostatistics, Memorial Sloan Kettering Cancer Center, New York, NY USA; 3 Brookdale Department of Geriatrics and Palliative Medicine, The Mount Sinai Hospital, Icahn School of Medicine at Mount Sinai, New York, NY USA; 4 Clinical Pharmacy Services, Department of Pharmacy, Memorial Sloan Kettering Cancer Center, New York, NY USA; 5 Adult Bone Marrow Transplant Service, Department of Medicine, Memorial Sloan Kettering Cancer Center, New York, NY USA; 6 Infectious Disease Service, Department of Medicine, Memorial Sloan Kettering Cancer Center, New York, NY USA

**Keywords:** Myeloma, Immunotherapy, Cancer immunotherapy

Dear Editor,

Bispecific antibodies (BsAbs) have demonstrated deep and durable responses in patients with relapsed/refractory multiple myeloma (RRMM) [1–3]. Three BsAbs are approved for RRMM treatment, including two targeting B-cell maturation antigen (BCMA), teclistamab and elranatamab, and one targeting G protein–coupled receptor class C group 5 member D (GPRC5D), talquetamab. While BsAbs are generally safe and well-tolerated, infectious complications are common during treatment [4–6].

Viral infections account for nearly half of all infections associated with BsAb therapy [4–6]. Cytomegalovirus (CMV) reactivation has been reported during BsAb therapy, but the incidence and risk factors associated with reactivation remain poorly defined [5]. A pooled analysis of 11 clinical trials of BsAbs for RRMM demonstrated CMV reactivation occurred in 8% of patients [6]. However, CMV testing was not routinely performed in these trials, therefore underestimating the true incidence and clinical significance of CMV reactivation during BsAb therapy [5, 6]. Two recent reports of RRMM patients treated with BsAbs, observed CMV reactivation rates as high as 49% [7, 8].

In this single-center study, we present real-world evidence of CMV reactivation in patients with RRMM receiving standard of care (SOC) BsAbs (teclistamab, elranatamab, or talquetamab) at Memorial Sloan Kettering Cancer Center (MSKCC) between November 1, 2022, and October 31, 2024. This study was approved by the MSKCC Institutional Review Board. A CMV surveillance program was implemented to identify CMV seropositive patients prior to BsAb treatment. Data were analyzed retrospectively.

CMV serostatus was determined by pretreatment serum CMV IgG. Patients with negative or unavailable CMV IgG testing were excluded. Serum CMV DNA was assessed via polymerase chain reaction (PCR) assay with a quantitation threshold of 34.5 international units per milliliter (IU/mL; COBAS® AmpliPrep/COBAS® TaqMan® CMV Test, Roche Diagnostics) [9]. Detectable CMV DNA at any level was defined as “DNAemia.” DNAemia above the quantitation threshold (≥34.5 IU/ml) was categorized as “CMV reactivation.” DNAemia below the quantitation threshold (<34.5 IU/ml) was categorized as “CMV detection.” Clinically significant CMV infection (csCMVi) was defined as CMV DNAemia treated with CMV-directed preemptive therapy or CMV disease with end-organ damage [10–12]. Repeat CMV PCR testing, surveillance, and treatment decisions were at the treating clinician’s discretion.

Descriptive statistics were used to summarize baseline patient and disease characteristics. Cumulative incidence was used to estimate both rates of CMV detection and CMV reactivation with initiation of new treatment or death as a competing event, starting from the time of BsAb initiation. Cause-specific analysis using the Cox proportional-hazard model was used to assess the association between risk of CMV and baseline risk factors, as well as determining the association between risk of CMV and each one of steroid-use and IVIG, as time-dependent covariates (TDCs). Additionally, CMV reactivation was used as a TDC when evaluating the association between CMV reactivation and both OS and PFS using the Cox proportional-hazard model. All analyses were performed using R 4.3.2 (R Core Team [13]).

In total, we identified 85 CMV seropositive patients (Table 1). Amongst these patients, 50 (59%) received teclistamab, 29 (34%) received talquetamab, and 6 (7.1%) received elranatamab. Additional patient characteristics are described in Table 1. All patients received herpes simplex and varicella zoster virus prophylaxis (ppx). IVIG ppx was given to 38/85 seropositive patients.

**Table 1:** Baseline patient characteristics.

Baseline characteristics	Total (*N* = 85)	CMV PCR negative (*N* = 70)	CMV PCR positive (*N* = 15)
**Median Age at BsAb Start**, **years (range)**	70 (51–84)	70 (51–84)	67 (55–81)
**Gender**			
Female	52 (61%)	39 (56%)	13 (87%)
Male	33 (39%)	31 (44%)	2 (13%)
**Race**			
Caucasian	51 (62%)	42 (63%)	9 (60%)
African American	19 (23%)	17 (25%)	2 (13%)
Asian	9 (11%)	6 (9.0%)	3 (20%)
Other	3 (3.7%)	2 (3.0%)	1 (6.7%)
**ECOG Performance Status**			
0	15 (31%)	14 (37%)	1 (9.1%)
1	32 (65%)	24 (63%)	8 (73%)
2	1 (2.0%)	0 (0%)	1 (9.1%)
3	1 (2.0%)	0 (0%)	1 (9.1%)
**R-ISS Stage**			
1	11 (22%)	10 (25%)	1 (10%)
2	34 (68%)	26 (65%)	8 (80%)
3	5 (10%)	4 (10%)	1 (10%)
**Cytogenetic Risk at Dx**			
Standard Risk	31 (47%)	25 (45%)	6 (55%)
High Risk^a^	27 (41%)	23 (42%)	4 (36%)
Ultra High Risk^b^	8 (12%)	7 (13%)	1 (9.1%)
**Cytogenetic Risk Prior to BsAb**			
Standard Risk	6 (14%)	5 (13%)	1 (17%)
High Risk	19 (43%)	17 (45%)	2 (33%)
Ultra High Risk	19 (43%)	16 (42%)	3 (50%)
**EMD at Diagnosis**	16 (22%)	12 (19%)	4 (33%)
**EMD within 3-Months of BsAb**	29 (39%)	23 (38%)	6 (43%)
**CNS Involvement**	2 (2.4%)	2 (2.9%)	0 (0%)
**Heavy Chain Isotype**			
IgG	59 (69%)	48 (69%)	11 (73%)
IgA	14 (16%)	13 (19%)	1 (6.7%)
IgD	1 (1.2%)	1 (1.4%)	0 (0%)
None	11 (13%)	8 (11%)	3 (20%)
**Light Chain Isotype**			
Kappa	50 (59%)	40 (57%)	10 (67%)
Lambda	33 (39%)	28 (40%)	5 (33%)
Biclonal	2 (2.4%)	2 (2.9%)	0 (0%)
**Median Prior LOT (range)**	6 (3-19)	6 (3-19)	7 (3–14)
**Prior Auto-SCT**	52 (63%)	43 (61%)	9 (69%)
**Prior Allo-SCT**	4 (4.8%)	4 (5.7%)	0 (0%)
**Prior BCMA-directed Therapy**	35 (42%)	30 (43%)	5 (38%)
**Prior CAR T-cell Therapy**	23 (28%)	21 (30%)	2 (15%)
**BsAb on Study**			
Teclistamab	48 (58%)	40 (57%)	8 (62%)
Elranatamab	6 (7.2%)	6 (8.6%)	0 (0%)
Talquetamab	29 (35%)	24 (34%)	5 (38%)
**Prior Detectable CMV**	15 (19%)	12 (18%)	3 (25%)
**Prior CMV Treatment**	4 (5.0%)	2 (2.9%)	2 (17%)
**Hemoglobin (g/dL)**			
Median (Q1, Q3)	9.35 (8.20, 11.10)	9.70 (8.40, 11.20)	8.40 (7.10, 9.50)
**Platelets (K/mcL)**			
Median (Q1, Q3)	133 (83, 193)	137 (94, 193)	109 (43, 176)
**ANC (K/mcL)**			
Median (Q1, Q3)	2.44 (1.50, 3.70)	2.50 (1.50, 3.70)	2.20 (1.90, 3.86)
**ALC (K/mcL)**			
Median (Q1, Q3)	0.85 (0.54, 1.40)	0.90 (0.60, 1.40)	0.71 (0.50, 1.20)
**IgG (mg/dL)**			
Median (Q1, Q3)	414 (275, 669)	433 (275, 687)	333 (278, 542)
**IgG** < **500** **mg/dL**	45 (62%)	36 (59%)	9 (75%)

*BsAb* Bispecific Antibody, *ECOG* Eastern Cooperative Oncology Group, *R-ISS* Revised International Staging System, *EMD* extramedullary disease, *CNS* Central Nervous System, *LOT* Lines of therapy, *BCMA* B-cell maturation antigen, *CAR* Chimeric Antigen Receptor, *ANC* Absolute neutrophil count, *ALC* Absolute Lymphocyte count.

^a^High Risk cytogenetics include 1q gain, t(4;14), t(14;16) or 17p deletion.

^b^Ultra High Risk cytogenetics include ≥2 high risk cytogenetic features.

**Fig. 1:**
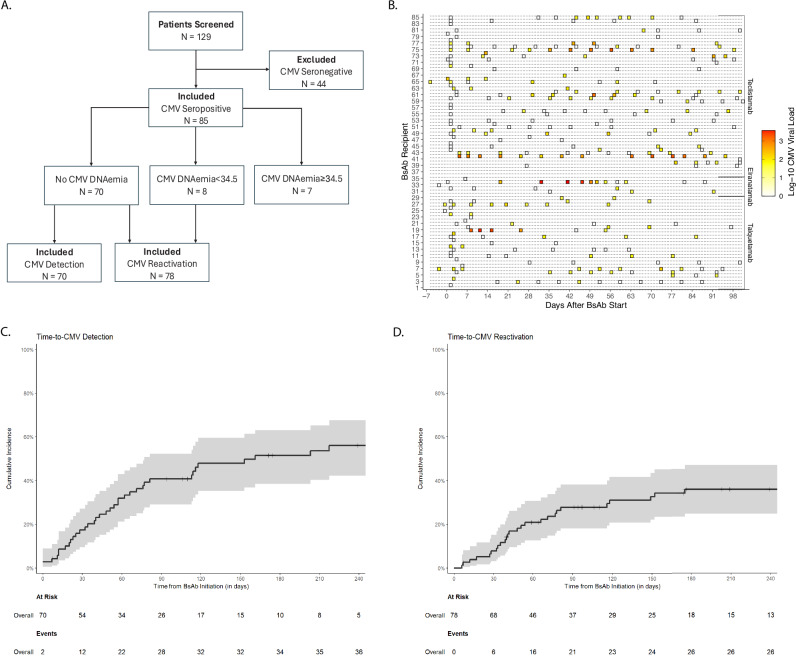
Incidence and Patterns of CMV Reactivation. **A** Schematic of patients included in study as well CMV detection and CMV reactivation analyses. **B** Heatmap of serum CMV DNA PCR during the first 100 days of BsAb treatment. Individual patients are depicted on the left Y-axis and grouped by BsAb depicted on the right Y-axis. Each box indicates an individual serum CMV DNA PCR test. Shading within the box corresponds to CMV DNV PCR log_10_ result. **C** Cumulative incidence of CMV detection and **D** reactivation during BsAb therapy. CMV Cytomegalovirus, PCR polymerase chain reaction, BsAb bispecific antibody.

Baseline PCR testing determined which patients were at risk of CMV detection and reactivation. Patients with baseline undetectable CMV PCR (*N* = 70) were considered at risk of CMV detection and reactivation whereas patients with baseline detectable CMV PCR < 34.5 IU/ml (*N* = 78) were only considered at risk of developing CMV reactivation (Fig. 1A). Patients with baseline CMV PCR ≥ 34.5 IU/ml (*N* = 7) met criteria for CMV reactivation prior to treatment thus were excluded from the CMV reactivation and detection analyses. A median of 4 (IQR, 1–13) PCR tests were ordered per seropositive patient throughout the follow-up period (Fig. 1B).

In the 70 patients at risk of CMV detection, estimated cumulative incidence of CMV detection was 41% (95% confidence interval [CI], 29–52%) at Day 90 and 51% (95% CI, 38–63%) at Day 180 (Fig. 1C). In the 78 patients at risk of CMV reactivation, estimated cumulative incidence of CMV reactivation was 28% (95% CI, 18–38%) at Day 90 and 36% (95% CI, 25–47%) at Day 180 (Fig. 1D). Overall, CMV reactivation occurred in 26 patients, of whom 6 developed CMV DNAemia >1000 (3 log_10_) IU/ml. Amongst these patients, 4/6 (67%) were treated with CMV-directed treatment. No patients with CMV DNAemia <1000 IU/mL received CMV-directed treatment.

Four patients developed csCMVi including one patient with CMV esophagitis and three patients with asymptomatic CMV DNAemia (peak CMV DNA PCR 1820.0-43700IU/ml). Preemptive therapy was started at a median 85.5 days (range 34–139 days) from the first BsAb dose. All four patients were treated with a BCMA-directed BsAb, three with teclistamab and one with elranatamab. Two patients, including the patient with CMV esophagitis, were treated with intravenous (IV) ganciclovir followed by oral valganciclovir. The other two patients with CMV DNAemia were treated only with valganciclovir. Symptoms and DNAemia resolved in all patients, though CMV reactivation recurred in two patients during gaps in anti-viral therapy. Only the patient with CMV esophagitis incurred a pause in BsAb therapy which was safely resumed once DNAemia resolved.

Baseline characteristics, including absolute lymphocyte count, absolute neutrophil count, hemoglobin, platelet count, and hypogammaglobulinemia (total IgG<500) were not associated with an increased risk of CMV reactivation.

Cytokine release syndrome (CRS) and tocilizumab treatment were not associated with an increased risk of CMV detection or reactivation. However, steroid treatment for CRS was associated with an increased risk of CMV detection (HR 3.11, 95%CI 1.25–7.74, *p* = 0.01) with a trend towards increased risk of CMV reactivation (HR 2.16, 95%CI 0.84–5.56, *p* = 0.10). Patients treated with IVIG did not have a lower risk of CMV detection or reactivation.

At a median follow-up of 11 months, CMV reactivation during BsAb treatment was associated with a worse OS (Kaplan-Meier, HR 3.34, 95%CI 1.17–9.52, *p* = 0.026) without significant difference in PFS. No patients died from csCMVi but death from other infections occurred in 7/10 (70%) patients with CMV reactivation compared to 2/8 (25%) patients without CMV reactivation. Fatal infections in patients with CMV reactivation included one death attributed to sepsis and six deaths attributed to pneumonia; COVID-19 (*N* = 3), RSV (*N* = 1), *Streptococcus pneumoniae* (*N* = 1), and *Pneumocystis jirovecii* (*N* = 1).

There are no guidelines for preemptive therapy in patients with CMV DNAemia though a threshold of 2–3 log_10_ IU/mL has been suggested in hematopoietic stem cell transplant recipients [10]. In our study, 4/6 patients with serum CMV DNA ≥ 3 log_10_ IU/mL received CMV-directed treatment. No patients with serum CMV DNA <3 log_10_ IU/mL were treated. Our observed rate of csCMVi is lower than other reports in which 25-48% of patients received preemptive therapy [7, 14]. Differences in preemptive therapy initiation may be explained by varying institutional guidelines. For example, Park et al. report an institutional preemptive therapy threshold of 500 IU/ml [7]. More data are needed to define an appropriate CMV DNAemia threshold for initiating preemptive therapy.

No baseline characteristics predicted the risk of CMV detection or reactivation. CMV detection or reactivation rate was comparable between BCMA-targeted vs. GPRC5D-targeted BsAbs though only patients treated with a BCMA-targeted BsAb were treated with CMV-directed therapy. This finding conflicts with other reports suggesting increased risk of CMV reactivation with BCMA-targeted BsAbs [7, 8]. This may be explained by different CMV reactivation thresholds and small sample size. A larger patient cohort is necessary to determine significant differences in CMV reactivation related to target antigen.

CRS was not associated with an increased risk of CMV detection or reactivation. However, steroid treatment for CRS increased CMV detection and may increase the risk of CMV reactivation. Similarly, a recent study reported an increased risk of CMV reactivation after chimeric antigen (CAR) T-cell therapy in patients treated with steroids for CRS [15]. Together these results suggest steroid treatment for CRS may be an important CMV reactivation risk factor associated with T-cell redirection therapies.

CMV reactivation was associated with worse OS. While CMV was not a documented cause of death, a higher proportion of patients with CMV reactivation died from other infections compared to those without CMV reactivation (70% vs 25%). Thus, CMV reactivation may reflect a severely immunocompromised state and serve as a harbinger of serious infectious complications.

To our knowledge, this is the largest real-world study of CMV reactivation in patients with RRMM treated with SOC BsAbs. Compared to other studies, we excluded patients treated on clinical trials as well as patients treated with combination therapies that may confer a higher risk of CMV reactivation [7]. Furthermore, patients were prospectively tested for CMV serostatus and CMV DNA PCR before BsAb therapy. However, repeat serum PCR testing and preemptive therapy were at the discretion of the treating clinician, which may have led to the underestimation of CMV reactivation and csCMVi during BsAb therapy. A prospective study with pre-planned CMV monitoring during BsAb treatment is needed to accurately estimate the incidence, clinical significance, and risk factors associated with CMV reactivation.

In conclusion, CMV reactivation is a common occurrence during BsAb therapy for RRMM. Surveillance is best reserved for CMV seropositive patients. We suggest weekly CMV surveillance in patients treated with steroids for CRS during the first month of treatment. Testing frequency may be adjusted depending on the presence and trend of CMV DNAemia but should continue for the first three months. Finally, the potential impact of CMV reactivation on clinical outcomes warrants further investigation.
